# Emergence dynamics of adult *Culicoides* biting midges at two farms in south-east England

**DOI:** 10.1186/s13071-022-05370-z

**Published:** 2022-07-11

**Authors:** Jessica Eleanor Stokes, Simon Carpenter, Christopher Sanders, Simon Gubbins

**Affiliations:** 1 The Pirbright Institute, Ash Road, Pirbright, Surrey GU24 0NF UK; 2 ProScience Ltd, Gloucestershire, GL11 5SD UK

**Keywords:** Arbovirus, *Culicoides* biting midges, Emergence Trapping, Long-term data, Surveillance, Vector-borne disease

## Abstract

**Background:**

*Culicoides* biting midges (Diptera: Ceratopogonidae) are biological vectors of livestock arboviruses that cause diseases with significant economic, social and welfare impacts. Within temperate regions, livestock movement during arbovirus outbreaks can be facilitated by declaring a ‘seasonal vector-free period’ (SVFP) during winter when adult *Culicoides* are not active. In this study we carry out long-term monitoring of *Culicoides* adult emergence from larval development habitats at two farms in the UK to validate current definitions of the SVFP and to provide novel bionomic data for known vector species.

**Methods:**

Standard emergence traps were used to collect emerging adult *Culicoides* from dung heaps at two cattle farms in the south-east of England from June to November 2016 and March 2017 to May 2018. *Culicoides* were morphologically identified to species or complex level and count data were analysed using a simple population dynamic model for pre-adult *Culicoides* that included meteorological components.

**Results:**

More than 96,000 *Culicoides* were identified from 267 emergence trapping events across 2 years, revealing clear evidence of bivoltinism from peaks of male populations of *Culicoides* *obsoletus* emerging from dung heaps. This pattern was also reflected in the emergence of adult female Obsoletus complex populations, which dominated the collections (64.4% of total catch) and emerged throughout the adult active period. Adult male *C. obsoletus* were observed emerging earlier than females (protandry) and emergence of both sexes occurred throughout the year. *Culicoides chiopterus* and *Culicoides scoticus* were also identified in spring emergence collections, providing the first evidence for the overwintering of larvae in dung heaps for these species.

**Conclusions:**

This study demonstrates continual and highly variable rates of emergence of *Culicoides* throughout the year. A lack of evidence for mass emergence in spring along with the ability to observe male generations highlights the need for complementary surveillance techniques in addition to light-trap data when investigating seasonality and phenology. Evidence was found of other vector species, *C. chiopterus* and *C. scoticus*, utilising cattle dung heaps as an overwintering habitat, further highlighting the importance of these habitats on farms.

**Graphical Abstract:**

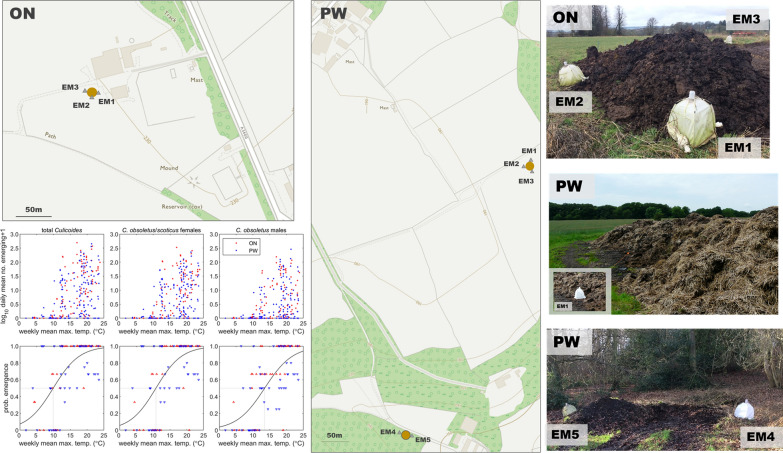

**Supplementary Information:**

The online version contains supplementary material available at 10.1186/s13071-022-05370-z.

## Introduction

The potential for increased emergence and spread of vector-borne diseases (VBDs) of medical and veterinary importance with changing environmental conditions is a global concern [1, 2]. VBDs are considered to be emerging at an increased rate, with 80% of the worlds’ human population considered currently at risk from at least one vector-borne disease [3, 4]. A recent white paper commissioned by the International Federation for Animal Health identified 31 important VBDs affecting humans and animals, and over half of these (16) were arboviruses [5].

*Culicoides* biting midges (Diptera: Ceratopogonidae) are biological vectors of veterinary and medical pathogens that cause diseases inflicting significant economic, social and welfare impacts on the livestock industry and human populations [6–9]. In ruminants, bluetongue virus (BTV) and Schmallenberg virus (SBV) in particular have been identified as important arboviruses, causing diseases with high, multifactorial associated costs during outbreaks in Europe [10–13]. In addition, following the emergence of BTV and SBV, the threat of successful emergence of other *Culicoides*-borne arboviruses is considered to be greater in Europe, particularly in the case of African horse sickness virus, which has caused a recent unprecedented epidemic in South East Asia [14] and historically has resulted in high equine mortality and substantial economic losses [15].

A key feature exacerbating the impact of both SBV and BTV in Europe is their ability to successfully overwinter in a region where *Culicoides* adult activity is limited for several months by cold temperatures that also prevent completion of the extrinsic incubation period [16–21]. Re-colonisation from warmer regions is the only primary mechanism underpinning overwintering of *Culicoides*-borne arboviruses in northern Europe identified to date and other potential methods are largely unknown and challenging to dissect, although both transplacental transmission [22] and longer-term survival of *Culicoides* in animal housing [23] have been identified.

Understanding the ecology of *Culicoides*-borne arboviruses in northern Europe is challenging and several fundamental aspects of vectorial capacity remain very poorly understood. This is in part due to the difficulties of studying what is thought to be a multi-vector system [24, 25], where none of the many candidate vector species have proved to be amenable to colonisation under laboratory conditions [26]. This issue is exacerbated by primary inference of ecology from light-suction trap collections where well-known biases exist, including variation in diurnal activity and potential light aversion following infection [27–29]. It is highly probable, however, from isolations and detection of viral RNA that the *Avaritia* subgenus (*Culicoides obsoletus* Meigen, *Culicoides scoticus* Downes & Kettle, *Culicoides dewulfi* Goetghebuer, *Culicoides chiopterus* Meigen and *Culicoides montanus* Shakirzjanova) and those within the *Culicoides* subgenus (*Culicoides pulicaris* Linnaeus, *Culicoides punctatus* Meigen) are primary vectors for BTV and SBV in northern Europe [30, 31].

*Culicoides* of the subgenera *Culicoides* and *Avaritia* are known to complete multiple generations during Spring–Autumn in northern Europe, followed by an overwintering period spent primarily in their fourth (final) larval instar stage [32]. Light-suction trap collections have been used to infer the number of generations completed each year, through either the appearance of large numbers of adult individuals over short time periods in light-suction [33] or un-baited suction traps [34]. Completion of gonotrophic cycles has also been inferred through abdominal pigmentation in adult female populations to complement trapping approaches [35, 36]. In addition, laboratory studies of the development of *C. obsoletus* in Spain have demonstrated that generation time from egg to pupation was approximately 32 days when derived from field populations held at a constant 18 °C [37]. In the UK it is inferred that species within the *Culicoides* and *Avaritia* subgenera are bivoltine or trivoltine [36, 38], although the timing and number of generations will clearly be directly related to biotic and abiotic parameters.

Larval development sites utilised by the *Culicoides* subgenus in northern Europe include marshland and swamp habitats, water body embankments, waterlogged areas, sheep dung and organically enriched soils [32, 39–42]. Those utilised by the *Avaritia* subgenus are often more closely associated with livestock farming. The most common and abundant livestock-associated species in northern Europe, *C. obsoletus*, demonstrates plasticity in larval development site selection, with individuals recovered from forest leaf litter, tree holes, flood plains, marshes, swamps and acid grassland habitats, silage residues, compost, fresh and stored manure of multiple species, livestock bedding and livestock housing [43–48]. *Culicoides scoticus* breeding habitats overlap with these and are similarly diverse [32, 42, 49, 50]. *Culicoides chiopterus* and *C. dewulfi*, in contrast, are considered obligate dung breeders [32, 40, 46, 51, 52]. Recent studies have widened the known larval habitats of these species, with some evidence of associations with floodplains and, in the case of *C. chiopterus*, compost, meadows, swampy forests and riverbanks [45].

The use of a diverse range of organically enriched breeding habitats by the *Avaritia* subgenus can lead to huge populations of these arbovirus vectors developing in close proximity to susceptible livestock hosts. The farming practice of storing dung and slurry onsite over the winter, prior to spreading in the spring, may further increase the risk of *Culicoides* emerging near livestock hosts both throughout the winter months and once spread in the spring [38, 42, 53]. Such habitats also represent stable, warmer, relatively permanent habitats for insects that are at risk of desiccation in more ephemeral substrates [54]. Conditions within dung heaps could extend emergence of adult *Culicoides* in northern Europe into winter, contributing to overwintering of arboviruses. Interestingly, despite these favourable developmental conditions, only *C. obsoletus* has been previously reported in high abundance emerging from cattle dung heaps [42, 54, 55]. Taking this into consideration, this study aimed to collect emerging *Culicoides* from dung heaps across multiple years and farm sites to investigate the emergence phenology and seasonality of these larval habitats for the informing of *Culicoides*-borne disease infection models, policy and control strategies.

## Methods

### Study area, *Culicoides* collection and identification

**Fig. 1:**
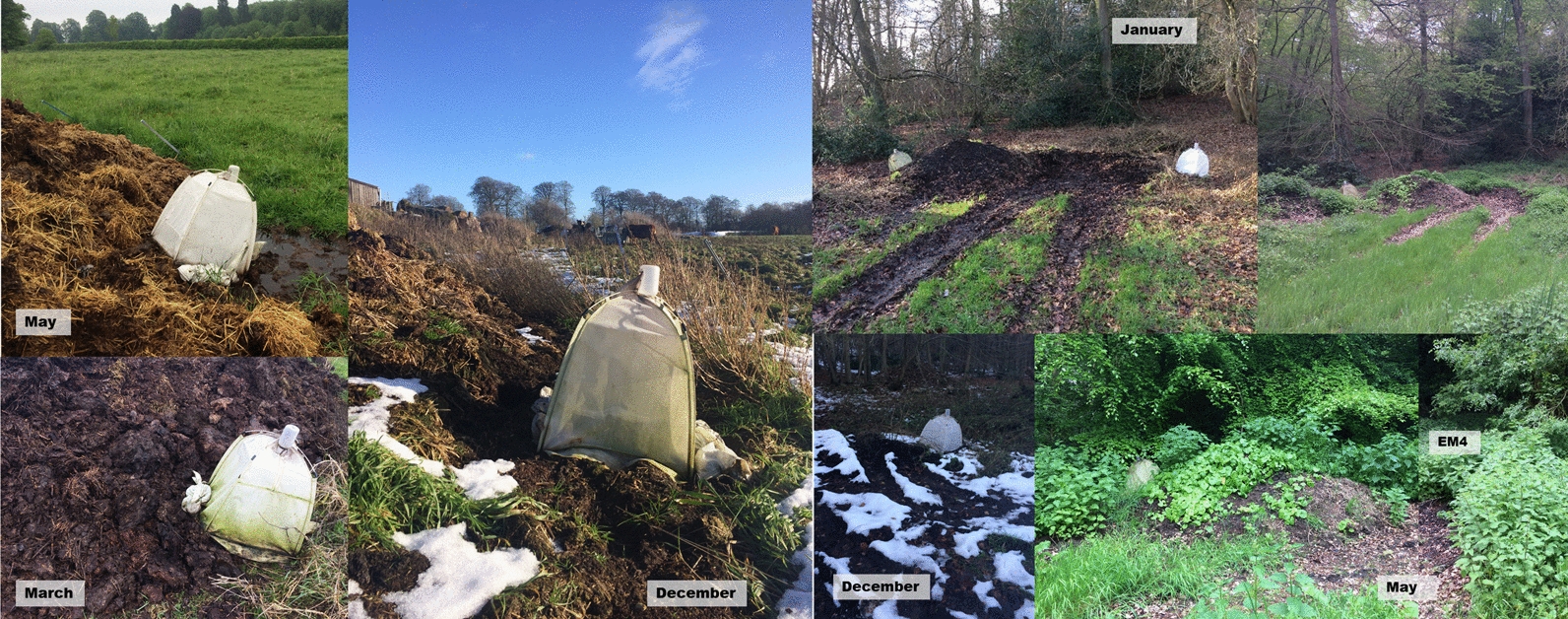
Locations of farm sites and dung heaps with corresponding emergence traps

**Fig. 2:**
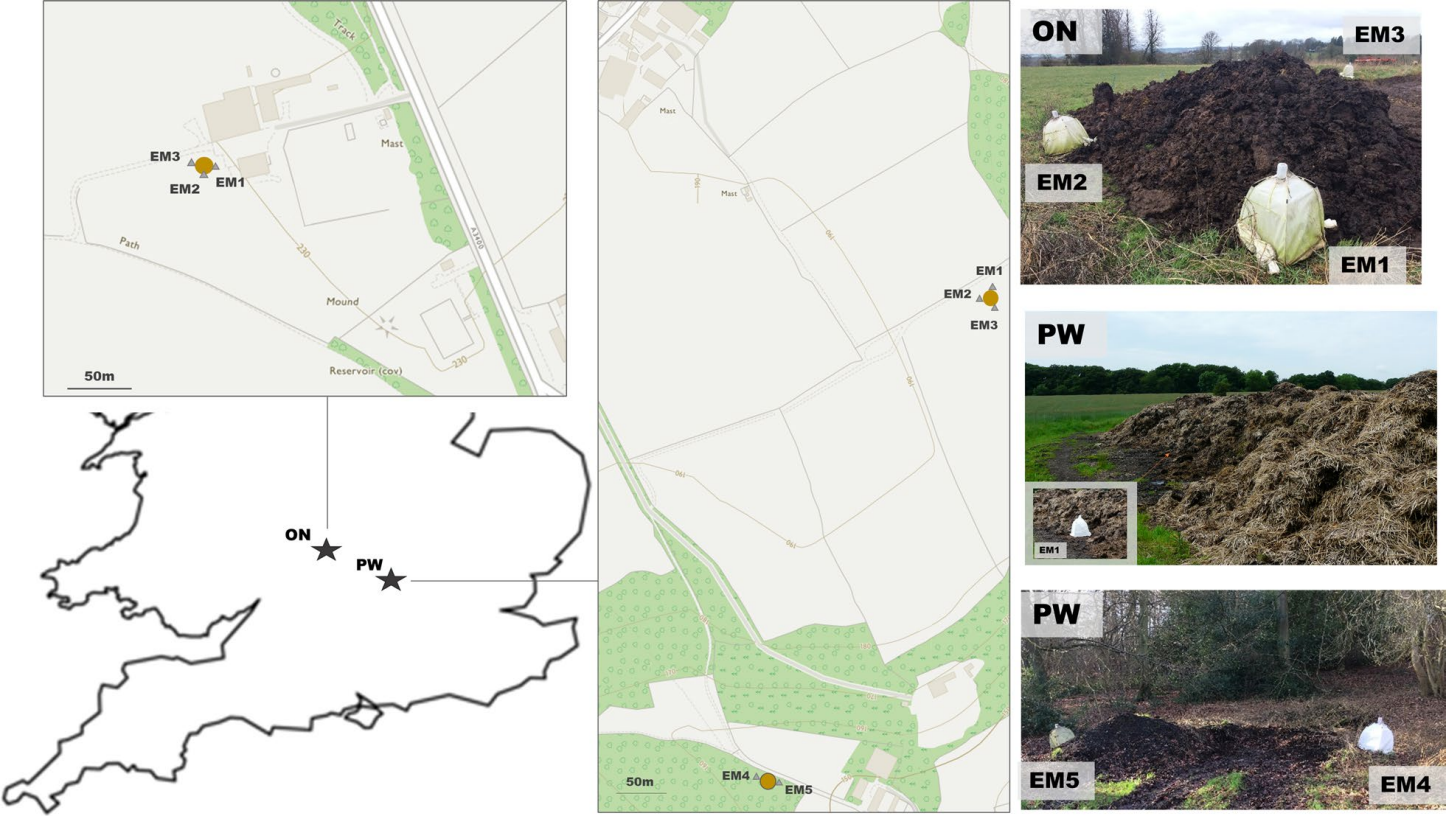
Example of site succession through the year at ON EM2 and PW EM4 and EM5

*Culicoides* were collected from two farms, ON (51.95, -1.52) and PW (51.69, -0.74), from 8 June 2016 to 8 November 2016 and from 14 March 2017 to 23 May 2018. At each site, emergence traps (NHBS, Totnes, UK) were placed on *Culicoides* larval habitats enclosing 0.36 m^2^ (three emergence traps at site ON; five at PW) (Fig. 1). The location of the traps did not change during the study; however due to vegetation succession over time the substrate did (Fig. 2). Newly emerged *Culicoides* were collected into 50–70% ethanol (a lower percentage of ethanol within this range was used during hot spells to reduce evaporation rate) and held in the collection bottle for up to 2 weeks prior to storage in fresh 70% ethanol. The daily collection rate was calculated by dividing total collection by number of trapping days between collections. Where mean daily collection rates were stated, these were the daily collection rate over the number of operational traps.

Adult *Culicoides* were identified to species or complex level using a Leica MZ6 stereo-microscope and morphological keys [32, 56]. Particularly large samples (> 4 g) were subsampled by draining excess ethanol and weighing, with a known proportion of the sample used for identification (samples are indicated in Additional file 7: Datafile S1). Female *Culicoides* of the *Avaritia* subgenus were identified to species level in the case of *C. chiopterus*, with *C. obsoletus* and *C. scoticus* females identified to Obsoletus complex level based on wing pattern morphology, pattern and size (examples of wing patterns can be viewed on the IKCC website [57]). *Culicoides montanus* was not included in the analysis as identification is only possible through molecular identification and has previously been considered rare and restricted to the southern Mediterranean region prior to 2020 [27, 58, 59]. Males of the *Avaritia* subgenus were identified to species level through morphological keys of the wings and genitalia [32, 56]. No formal description of *C. montanus* males exists to the authors' knowledge. The ratio of *C. obsoletus* and *C. scoticus* males was used as a proxy for the species ratio of females within the Obsoletus complex [34, 60, 61]. All female *Culicoides* were further examined for abdominal pigmentation, noted as non-pigmented, pigmented, gravid and blood-fed [62].

Hourly meteorological data for 2016, 2017 and 2018 were obtained from the Met Office for the weather stations closest to the trap sites (South Newington for ON; High Wycombe for PW; both located within 5 km of the traps). These were used to compute daily mean and maximum air temperatures (°C) and daily total precipitation (mm) (Additional file 2: Figure S1). In addition, hourly soil temperature data (at a depth of 3.5 cm) for the same years were extracted for each trap site from the ERA5-Land hourly data from 1950 to present [63].

### Statistical methods

The relationship between emergence of *Culicoides* biting midges and the mean maximum air temperature (°C) in the preceding week was assessed using a binomial family generalised linear mixed model. The response variable was whether or not an emergence trap collected any individuals; the mean maximum air temperature in the preceding week was the explanatory variable and trap was included as a random effect. Separate models were constructed for total *Culicoides*, *C. obsoletus/scoticus* females and *C. obsoletus* males. Models were implemented using the lme4 package [64] in R (version 4.0.5) [65].

The emergence trap data were further analysed using a simple population dynamic model. Because there is no information on the development of separate life stages (i.e. eggs, larvae and pupae), the model considers all pre-adult *Culicoides* life stages as a single population. The model assumed that: (i) the number of new pre-adults is proportional to adult *Culicoides* activity; (ii) pre-adult survival is density dependent; (iii) pre-adult development is temperature dependent at a rate, *d*, proportional to temperature above a threshold, *T*_min_ (i.e. the development rate is given by *δ* = *d*(*T*-*T*_min_) where *T* is the soil temperature), so pre-adults emerge once they have accumulated sufficient thermal time; (iv) there is no diapause. Parameters in the model were fit to the emergence trap data using Bayesian methods. Full details of the model and fitting methods are provided in the supporting information (Additional file 1: Text S1). Separate models were fitted for total *Culicoides*, *C. obsoletus/scoticus* females and *C. obsoletus* males.

## Results

### Culicoides collections

**Fig. 3:**
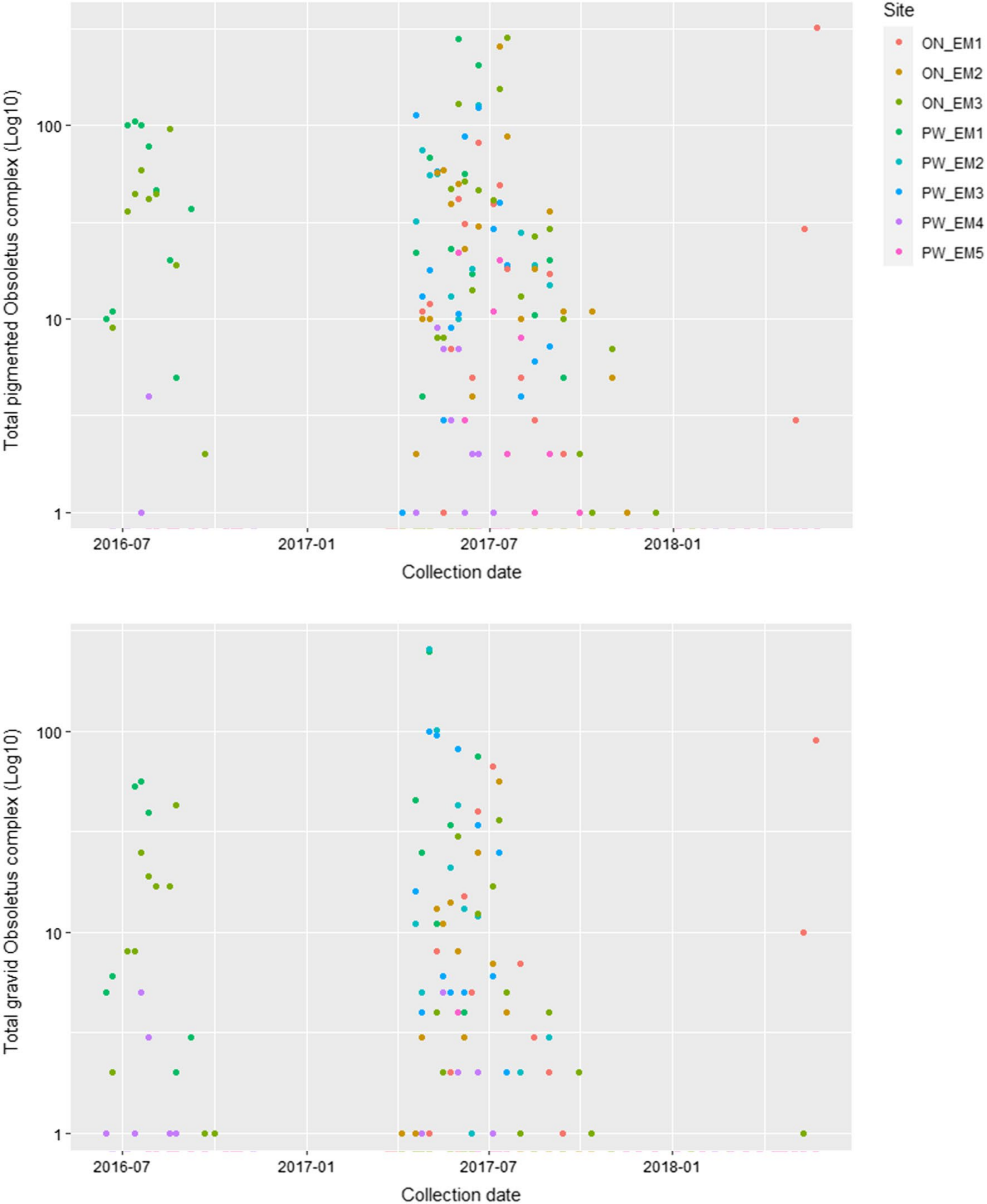
Total collected pigmented (top) and gravid (bottom) *Culicoides obsoletus/scoticus* collected from all emergence traps across both ON and PW farm sites

In total 286 collections were made during the study; however, 19 were not used in analyses because of disturbance of the traps by humans, livestock and/or wildlife, or due to extreme wind conditions leading to traps becoming untethered from the substrate. A total of 96,062 *Culicoides* belonging to seven species were collected across these 267 successful collections (Table 1). The collections analysed were dominated by females classified within the Obsoletus complex (61,852, 64.4%) and male *C. obsoletus* (33,453, 34.8%) (Table 1). Based on the ratio of male *C. obsoletus:C. scoticus*, 98.4% of these females were *C. obsoletus*. The sex ratio in emerging *Culicoides* was 1.82:1 female:male for the Obsoletus complex. Among the 61,852 female Obsoletus complex *Culicoides* collected, 5042 (8.2%) possessed abdominal pigmentation and 2263 (3.7%) were gravid. Both pigmented and gravid individuals were recovered throughout the study period (Fig. 3).

**Table 1:** Total *Culicoides* collected from each emergence traps

Trap	Female Obsoletus complex	Male *C. obsoletus*	Male *C. scoticus*	Female *C. chiopterus*	Male *C. chiopterus*	Other species	Total
ON EM1	9056	3162	88	1	1	1	12,309
ON EM2	10,444	5901	12	50	0	0	16,407
ON EM3	15,431	7083	233	11	17	30	22,805
PW EM1	13,808	9984	115	16	1	0	23,935†
PW EM2	3694	2548	19	0	0	0	6261
PW EM3	3290	2109	44	0	0	1	5444
PW EM4	3316	1954	17	1	0	70	5358
PW EM5	2813	712	4	5	0	7	3543‡
Total	61,852	33,453	532	84	19	109	96,062

No *Culicoides dewulfi* were trapped in any emergence trap

Other species:

ON EM1: 1 female *C. brunnicans*

ON EM3: 5 female *C. pulicaris*, 13 male *C. pulicaris*, 7 female *C. punctatus*, 2 male *C. punctatus*, 2 female *C. circumscriptus*, 1 female *C. brunnicans*

PW EM3: 1 female *C. pulicaris*

PW EM4: 39 female *C. pulicaris*, 30 male *C. pulicaris*, 1 female *C. brunnicans*

PW EM5: 6 female *C. pulicaris*, 1 male *C. pulicaris*

^†^ PW EM1: Total includes 11 damaged male Obsoletus group *Culicoides*

^‡^ PW EM5: Total includes two damaged male Obsoletus group *Culicoides*

**Table 2:**
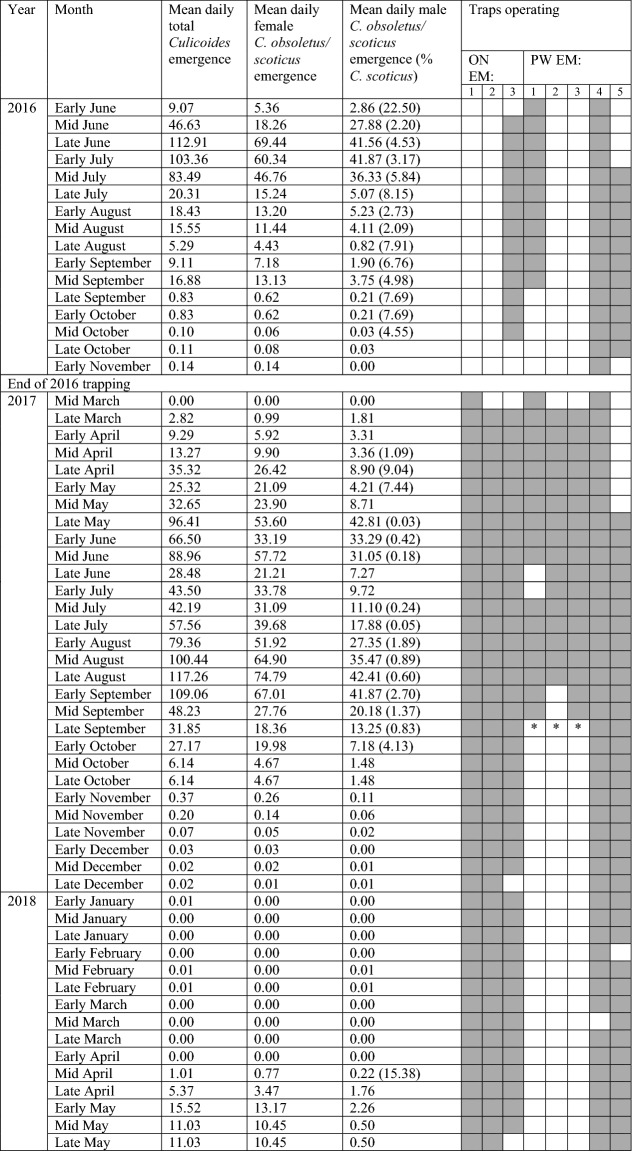
Daily rate of total *Culicoides* emergence, female *C. obsoletus/scoticus* emergence and male *C. obsoletus/scoticus* emergence across the study

Mean daily emergence rate is the number emerging divided by number of days each trap was running, across the number of traps.

Shaded squares demonstrate traps collected from during the period. Gaps in data in 2017/2018 represent trap damage caused by strong winds and/or interference from livestock/wildlife preventing complete collection.

*Dung heap removed in line with farmers practices

**Fig. 4:**
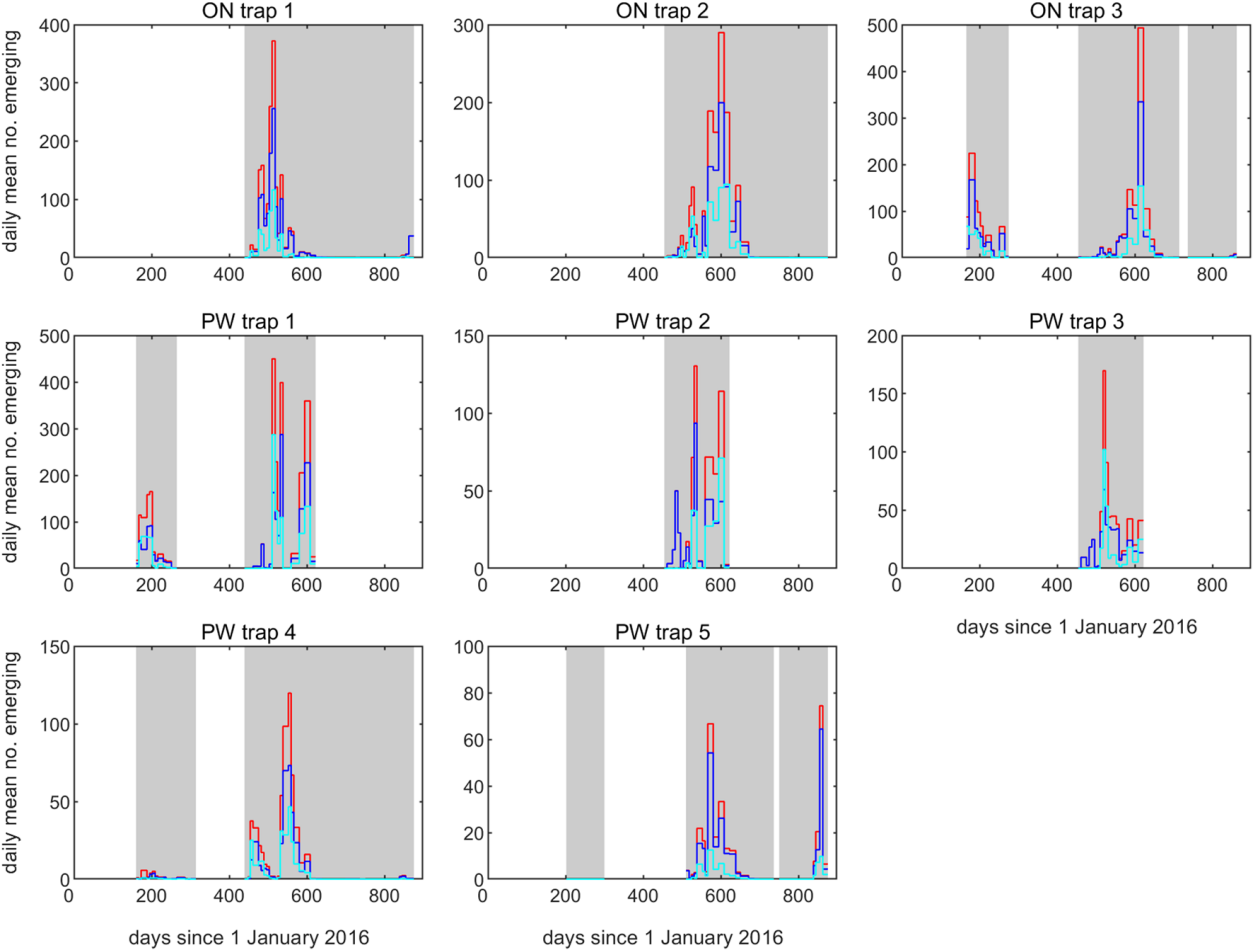
Daily number of *Culicoides* biting midges caught in eight emergence traps at two farm sites in south-east England during 2016–2018. Each panel shows the daily means for the total number of *Culicoides* emerging (red), the number of *Culicoides obsoletus/scoticus* females emerging (blue) and the number of *Culicoides obsoletus* males emerging (cyan). The grey-shaded areas indicate when the samples were being collected from the trap

Total recorded *Culicoides* abundance was greatest mid-August to early September 2017, recording a peak mean daily rate in late August of 117.3 (*SD* = 3.9) *Culicoides* emerging each trap day across the eight traps. This was also observed for the female Obsoletus complex (*M* = 74.5, *SD* = 3.7); however, *C. obsoletus* males peaked in both late May 2017 (*M* = 42.8, *SD* = 19.0) and then again in late August/early September 2017 (*M* = 42.4, *SD* = 0.6, *M* = 41.9, *SD* = 0.5, respectively) (Fig. 4; Table 2). *Culicoides scoticus* males were significantly less abundant, with peak daily emergence observed in late June 2016 (*M* = 1.9, *SD* = 0.0) and early September 2017 (*M* = 1.1, *SD* = 0.0). Other species were less abundant, with 103 *C. chiopterus* emerging sporadically from June to August 2016, June to late September 2017 and in late April/early May in 2018. The 95 *C. pulicaris* collected emerged from mid-June to late August 2016 and mid-May 2018. One gravid female was caught during early April 2017, suggesting the individual was preparing to oviposit on the dung heap. All nine *C. punctatus* were collected in June, seven of which from a single catch at ON EM3 in June 2016. The three *Culicoides brunnicans* Edwards were collected in mid-June 2016 and mid-May 2017, with both *Culicoides circumscriptus* Kieffer individuals recovered from ON EM3 in late August/early September 2016.

A major emergence event occurred at ON EM3 between 30 August and 12 September 2017 with a total of 6900 *Culicoides* collected (a daily rate of emergence of 492.9), with greatest total recorded abundance observed mid-August to early September in 2017 (Table 2). This is in contrast to 2016 where total recorded abundance was greatest in late June through to mid-July (mean daily rate of 99.9 *Culicoides* emerging/day across 3 running traps, *SD* = 17.3), with only 5.3 *Culicoides* emerging per trap day in late August 2016 (Fig. 4; Table 2). The mean daily *Culicoides* collection rate per trap varied from 0 to 492.9 with PW EM1 the most productive trap: collecting a mean of 92 *Culicoides* per collection across the study. The mean daily collection rate varied from 0 to 119.1 individuals across successful collections made in the study.

In 2016–2017 trapping ceased prior to the end of *Culicoides* emergence (Table 2). However, on restarting the traps no Culicoides were collected from 14 March to 22 March 2017. In the 2017–2018 trapping period, no female Culicoides emerged from the 5 January 2018 to 23 April 2018. Male emergence paused between the 5 January 2018 and 12 February 2018, when one male *C. obsoletus* emerged (between 12 February and 27 February), with no male *Culicoides* observed after this until 12 April 2018. From 8 June 2016 to 8 November 2016, 23 March 2017 to 4 January 2018 and after 12 April 2018 to the end of the study, emergence was observed to be continuous but the number of individuals collected was highly variable (Table 2).

The SVFP as defined by EU legislation and recorded in parallel in the UK using light-suction trap data was declared to be between 20 December 2016 to 15 April 2017 and from 7 December 2017 to 27 April 2018 (Dr Marion England, UK *Culicoides* Reference Laboratory, personal communication, June 17 2021, unpublished data). A total of 1185 *Culicoides* emerged from 23 March to 15 April 2017 (no emergence trapping was conducted over the winter prior to the 14 March 2017; Table 2) and 216 *Culicoides* emerged between 7 December 2018 and 27 April 2018, with the majority of these insects emerging within 2 weeks of the end of the vector-free period (78.0% and 98.2%, respectively).

### Relationship between emergence and temperature

**Fig. 5:**
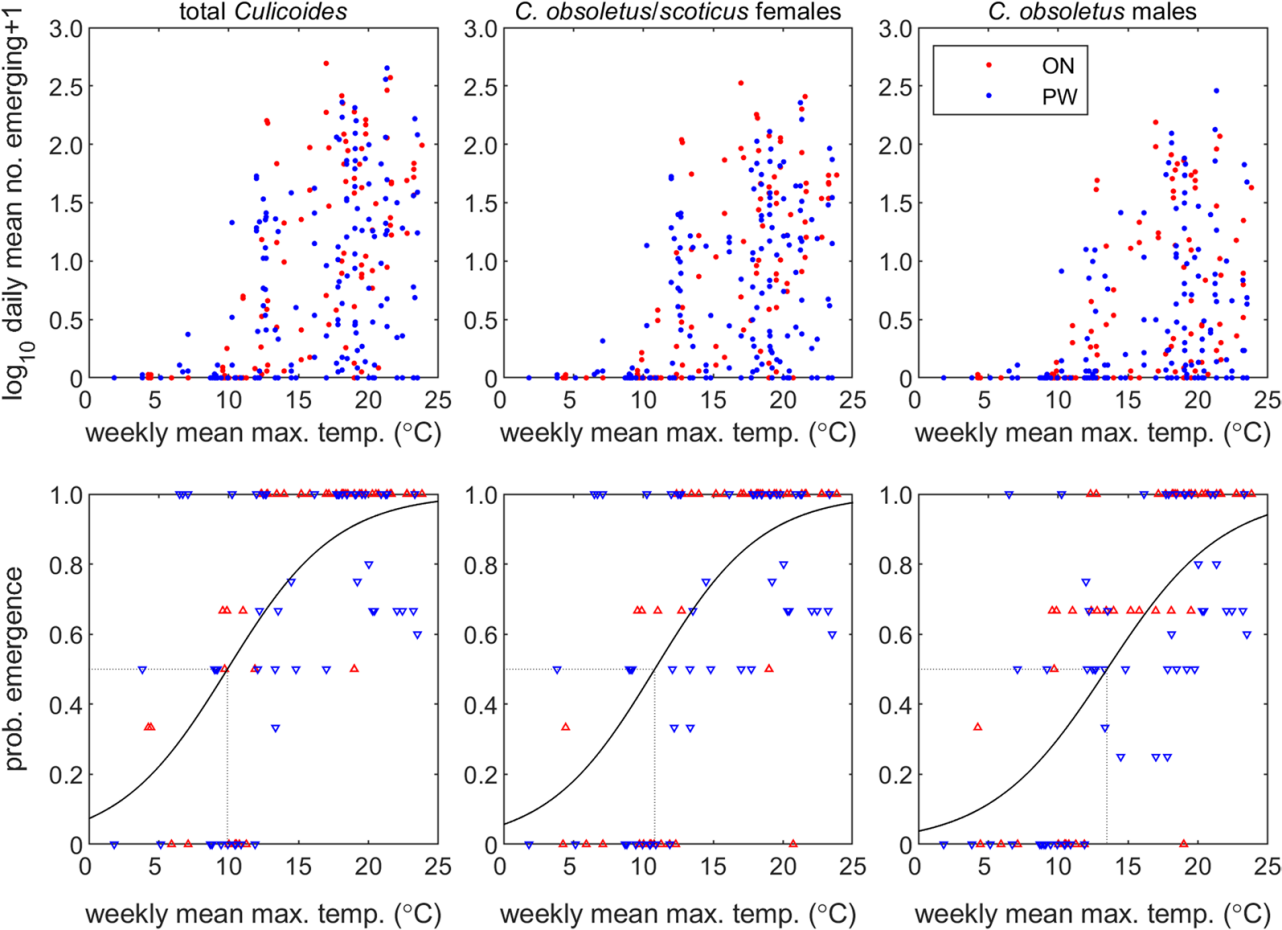
Relationship between emergence of *Culicoides* biting midges and the mean maximum temperature (°C) in the preceding week. The top row shows the daily mean number of midges emerging at each temperature. The bottom row shows the proportion of emergence trap catches with at least one midge at each temperature (triangles) and the estimated probability of emergence (black line). Columns show emergence for total *Culicoides* (left), *Culicoides obsoletus/scoticus* females (middle) and *Culicoides obsoletus* males. Colour indicates site: ON (red) or PW (blue)

Emergence increased with the mean maximum air temperature in the preceding week, in terms of both numbers emerging and the probability of emergence (i.e. the proportion of traps in which any midges were caught) (Fig. 5). The mean maximum air temperature at which 50% of traps contained at least one midge was 9.9 °C for total *Culicoides*, 10.8 °C for *C. obsoletus/scoticus* females and 13.5 °C for *C. obsoletus* males (Fig. 5).

### Number of generations

The number of generations was inferred from the data by defining separate peaks of emergence within a year. For 2016 and 2018 the limited data made it difficult to discern generation number (Fig. 4). However, the generations can be clearly identified in 2017, with one or two generations at each trap location (Fig. 4). At ON there were two generations peaking around day 150 (late May) and day 250 (early September). However, only the peak at day 150 was seen at ON EM1 and only the peak at day 250 was seen at ON EM3. By contrast, at ON EM2 both peaks were observed, though the one at day 150 was much smaller than the one at day 250 (Fig. 4). At PW EM1 and EM2 there were two generations of equal-size peaking at days 150 and 225. Two generations were also observed at PW EM4, though in this case there was a small peak at day 100 and a larger peak at day 180. Only one generation was seen at PW EM3 (peaking at day 160) and PW EM5 (peaking at day 200; though an earlier peak could have been missed at this location due to timing of sampling). Based on the time between peaks, these results suggest a generation time for the summer generation Obsoletus complex of around 75–80 days.

In most cases, the total numbers of *Culicoides* emerging, the number of Obsoletus complex females emerging and the number of *C. obsoletus* males emerging all had peaks at the same times, though of different heights (Fig. 4). However, at PW EM2, and to a lesser extent at EM3, there was a peak of emergence of Obsoletus complex females around day 120 without a corresponding emergence of males (Fig. 4).

### Simple population dynamic model for pre-adult Culicoides

The fitted models are shown in Additional file 3: Figure S2, Additional file 4: Figure S3 and Additional file 5: Figure S4 and parameter estimates presented in Additional file 6: Table S1. This suggests that differences in the number of adults emerging into each trap are primarily due to a difference in productivity in the underlying substrate (which varies amongst traps) rather than due to differences in density-dependence (which is similar amongst traps) (Additional file 6: Table S1). Furthermore, productivity was most similar for traps on the same dung heaps (ON EM1-3, PW EM1-3 and PW EM4-5) (Additional file 6: Table S1).

**Fig. 6:**
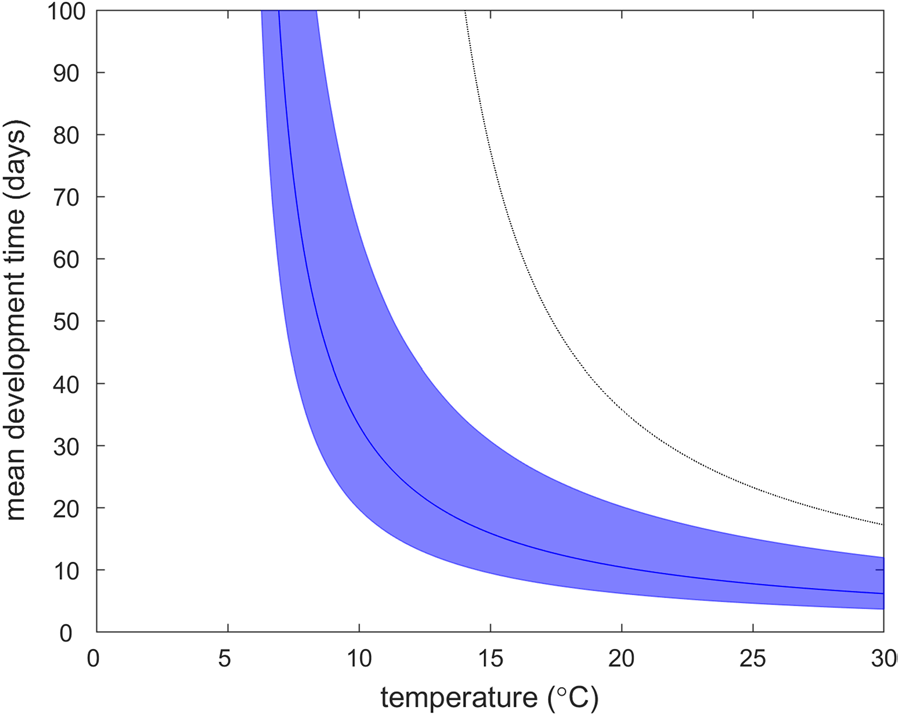
Development time for pre-adult *Culicoides* and its dependence on temperature inferred from the emergence trap data. The plot shows the posterior median (blue line) and 2.5th and 97.5th percentiles (blue shading) for the mean development time at each temperature. The black dotted line indicates the mean development time estimated in the laboratory for *Culicoides sonorensis* [83, 84]

Fitting the model also allowed the temperature-dependent development time to be estimated (Fig. 6). The amount of thermal time required for development (1/*d*)) was estimated (posterior median and 95% credible intervals) to be 151.5 (89.8, 290.7) day-degrees, with a threshold temperature for development (*T*_min_) of 5.4 (5.2, 5.6) °C (Additional file 6: Table S1). The corresponding mean development times are 21.4 (19.8, 64.4) days at 10 °C, 6.7 (6.2, 20.2) days at 20 °C and 4.0 (3.7, 12.0) days at 30 °C (Fig. 6). Furthermore, the estimated development rates were the same when fitting the model to emergence data for total *Culicoides*, *C. obsoletus/scoticus* females and *C. obsoletus* males (Additional file 6: Table S1).

## Discussion

While extremely intensive, multi-country studies have used light-suction trapping to define spatial and temporal peaks in adult *Culicoides* population abundance in northern Europe [59, 66], this study is to our knowledge the first to use emergence trapping of adults systematically across multiple locations and years to accomplish this aim. The collection of > 96,000 *Culicoides* from 267 emergence trapping events across 2 years revealed clear evidence of bivoltinism from peaks of both female Obsoletus complex populations and male populations of *C. obsoletus* emerging from dung heaps. This pattern has been reflected in daily suction trap data when collecting at height without lures [34], but was not reflected in light-suction trapping data used for surveillance purposes in the UK [36]. This is likely due to the biased nature of the light lure in such traps and subsequent limited numbers of male *Culicoides* usually collected using that method [67–69]. Despite this, it was clear that, in the habitats sampled, emergence of adult *Culicoides* during the winter period (November–April) was negligible, correlating with the wider light-suction surveillance trapping network in England and previous estimates of seasonality [34].

It has been reported previously that *Culicoides* undergo a ‘spring flush’, where a mass adult emergence event occurs in spring at the beginning of the vector season, coinciding with favourable weather conditions [34, 36, 39, 70–73]. This phenomenon has been widely reported in light-suction trap data; however, the data presented both here and in other early-season emergence studies demonstrate that adult appearance starts steadily. This apparent disparity is likely a function of meteorological conditions limiting flight as the previously reported threshold for adult flight activity is similar to that of adult emergence reported here [74]. Spring activity may therefore be better described as a mass host-seeking event under suitable conditions rather than reflective of synchronised emergence.

Dung heaps provide overwintering habitats for *Culicoides* as warm, permanently moist microclimates enabling continuous development and low-level emergence during colder months [42, 54, 71]. Indeed, despite soil conditions during this study being on average only 0.6 °C (99% range: − 3.3 to 4.3) warmer than ambient air temperature, core dung heap internal mean temperatures are 30 °C warmer than mean ambient air temperatures [75]. The degree to which *Culicoides* are exposed to these extremely divergent temperatures appears limited, however, as the Obsoletus complex generation time observed, indicated by corresponding peaks in emergence around late spring and early autumn, was longer than previously reported at 75–80 days [36, 38]. Studies reporting shorter generation times have previously been observed, and an additional peak has been identified in emergence in the summer, which was not observed here. This could be due to the nature of the developmental habitat (i.e. dung heaps become less attractive over late spring/early summer than other ephemeral habitats) or due to other undefined factors. The simple population dynamic model demonstrated similar emergence per heap, with differences between heaps due to individual heap productivity. This further points to the viability of dung heaps as developmental habitats for Obsoletus group *Culicoides.*

Male *Culicoides* represented a larger proportion of the overall catch earlier in the season, with male *C. obsoletus* first to emerge in 2018, providing additional evidence of protandry in *C. obsoletus*. This is similar to reports from emergence trap data for *C. obsoletus*, *C. chiopterus* and *C. dewulfi* [51], light-suction trap data for *Culicoides impunctatus* Goetghebuer [70] and anecdotal observations for *Culicoides imicola* Kieffer [76]. This suggests the reproductive strategy of earlier male emergence to be potentially widespread within *Culicoides*, as found in other insect species [77].

Biased sex ratios in studies of *Culicoides* emergence have been reported towards both female [39, 44, 70, 78] and male forms [38, 47]. It has been hypothesised that this is driven by selective larva mortality between the sexes, with faster development being advantageous with higher water content (hypothesised to favour male emergence) and higher decaying organic content being advantageous to longer developmental times (favouring female emergence) [38]. The female skew of emergence reported here, particularly with ongoing decomposition and succession at the dung heaps, adds weight to this hypothesis. Further work would be needed to truly determine the drivers of sex ratio as the impact of temperature and diet will vary significantly both spatially and temporally in the dung heaps, potentially influencing the patterns of emergence of the sexes as observed here [37].

Previous authors have noted the presence of abdominal pigmentation within adults collected through emergence trap collections and questioned the reliability of parity determination through this technique [42]. This study also observed pigmented individuals within the traps, at a rate of 8.2% of Obsoletus complex females. Pigmented individuals could be older nulliparous females exhibiting pigmentation as noted for *C. imicola* [79] and discussed further by Harrup et al. [42], suggesting an influence of dietary components associated with organically enriched substrates that should be further explored. The presence of a substantial number of gravid individuals (3.7% of adult *Culicoides* collected) was more surprising, however, and remains to be explained. Autogeny has been reported for other *Culicoides* species, most notably *C. impunctatus* [70, 80]; however, this has not been demonstrated for *C. obsoletus* to date, and this phenomenon has not been recorded in any major arbovirus vector species within the genus [7].

Numerous previous studies have found *C. scoticus* to co-exist in known *C. obsoletus* breeding habitats [32, 39, 42, 50, 81, 82]. This was also the case in the present study, with emergence of *C. scoticus* males observed throughout, including early in the season. This suggests overwintering within the dung heaps and further demonstrates that *C. obsoletus* and *C.* *scoticus* overlap significantly in their larval ecology. To our knowledge, this is the first evidence of male *C. scoticus* emerging from dung heaps, having utilised the habitat for overwintering [42, 44, 52, 82].

Other species of *Culicoides* caught made up 0.23% of the overall catch and occurred sporadically over the summer months, suggesting the use of dung heaps as a temporary summer breeding habitat by these species. The exception to this was one gravid female *C. pulicaris*, retrieved from a collection in April 2017, and unpigmented female *C. chiopterus* collected in late April/early May 2018. Although *C. chiopterus* has been previously reared from cow dung and observed in emergence traps on dung heaps [40, 45, 51, 82], this, to the authors' knowledge, is the first evidence of the use of cattle dung heaps in the overwintering of this species.

## Conclusions

This study has demonstrated the continual and highly variable emergence of *Culicoides* throughout the year from a semi-permanent larval habitat. We observed no evidence for mass emergence in spring, previously suggested to underpin adult activity patterns. This and the ability to observe seasonal patterns of emergence in male *C. obsoletus* highlight the need for complementary surveillance techniques in addition to light-trap data when investigating seasonality and phenology. Evidence was found of other vector species, *C. chiopterus* and *C. scoticus*, utilising cattle dung heaps as an overwintering habitat, further highlighting the importance of these habitats on farm. Light suction trap collection data should not be used as a proxy to infer generation or emergence of *Culicoides*, as adult flight activity may not reflect emergence activity.

## Supplementary Information

**Additional file 1: Text S1.** Simple population dynamic model for pre-adult *Culicoides* biting midges

**Additional file 2: Figure S1.** Air and soil temperature data for 2016–2018 for each farm site: daily mean and maximum air temperature (°C) (top and middle rows, respectively) and daily mean soil temperature at a depth of 3.5 cm (bottom row) for ON (left-hand column) and PW (right-hand column). Line colour indicates the year: 2016 (red); 2017 (blue); or 2018 (cyan).

**Additional file 3: Figure S2.** Observed and expected daily number of *Culicoides* biting midges caught in eight emergence traps at two farm sites in south-east England during 2016–2018. Each panel shows the observed daily mean (red) and the posterior median (blue dots) and 95% credible interval (shading) for the expected daily mean. The grey-shaded areas indicate when the samples were being collected from the trap

**Additional file 4: Figure S3.** Observed and expected daily number of *Culicoides obsoletus/scoticus* females caught in eight emergence traps at two farm sites in south-east England during 2016–2018. Each panel shows the observed daily mean (red) and the posterior median (blue dots) and 95% credible interval (shading) for the expected daily mean. The grey-shaded areas indicate when the samples were being collected from the trap.

**Additional file 5: Figure S4.** Observed and expected daily number of *Culicoides obsoletus* males caught in eight emergence traps at two farm sites in south-east England during 2016–2018. Each panel shows the observed daily mean (red) and the posterior median (blue dots) and 95% credible interval (shading) for the expected daily mean. The grey-shaded areas indicate when the samples were being collected from the trap

**Additional file 6: Table S1.** Parameter estimates for a simple population dynamic model of pre-adult *Culicoides* biting midges.

**Additional file 7:** **Data S1. **Full data set of collected *Culicoides* including site, trap location, collection date and morphological identification

## Data Availability

The dataset supporting the conclusions of this article is included within the article (and its additional files). The emergence trapping methodology has been included on the Gnatwork website. The Gnatwork is a Global Challenges Research Funded network with an international community of members researching *Culicoides* biting midges, sandflies and blackflies. Membership is free, with open access to resources through the website: https://www.gnatwork.ac.uk
