# Supplementary material for: Emergence dynamics of adult Culicoides biting midges at two farms in south-east England
Source: Parasit Vectors. 2022 Jul 11;15:251. doi: 10.1186/s13071-022-05370-z (PMC9277857; doi:10.1186/s13071-022-05370-z)
Supplement: Supplementary file 1 — Additional file 1: Text S1. Simple population dynamic model for pre-adult Culicoides biting midges [file 13071_2022_5370_MOESM1_ESM.docx]

**Text S1.** **Simple population dynamic model for pre-adult *Culicoides* biting midges**

The emergence trap data were analysed using a simple population dynamic model. Because there is no information on separate life stages (i.e. eggs, larvae and pupae), the model considers all pre-adult *Culicoides* life stages as a single population. The model assumes that: (i) the number of new pre-adults is proportional to adult *Culicoides* activity; (ii) pre-adult survival is density dependent; (iii) pre-adult development is temperature-dependent at a rate proportional to temperature above a threshold, so pre-adults emerge once they have accumulated sufficient thermal time; and (iv) there is no diapause.

**S1.1 Model equations**

Making these assumptions the expected number of pre-adults in substrate under trap *i* on day *t*+1 is given by

where *f_i_* indicates the farm site on which trap *i* is located and Δ*t*=1 is the daily time step. The first term in equation gives the number of pre-adults surviving to the next day that do not complete their development. The daily probability of survival is given by

$$s_{i}(t)=\frac{1}{1+\alpha_{i}L_{i}(t)^{\beta_{i}}},$$

where *α_i_* and *β_i_* are trap-specific density-dependence parameters. The daily development rate at farm site *f* is given by

$$\delta_{f}(t)=\left\{\begin{matrix} 0 & T_{S}^{(f)}(t)<T_{\min}, \\ d(T_{S}^{(f)}(t)-T_{\min}) & T_{S}^{(f)}(t)\geq T_{\min}, \end{matrix}\right.$$

where *d* is the development rate, *T*_min_ is the threshold temperature for development and *T_S_* is the daily mean soil temperature at a depth of 3.5 cm. The second term in equation gives the number of new pre-adults (i.e. eggs laid by adult females) where *ϕ_i_* is a measure of the productivity of the substrate under trap *i* and

$$A_{f}(t)\propto\exp\left(\sum_{n=1}^{2} a_{n}^{(f)}\sin\left(\frac{2\pi n}{365}t\right)+b_{n}^{(f)}\cos\left(\frac{2\pi n}{365}t\right)+c_{f}T_{A}^{(f)}(t)\right),$$

is the adult activity for farm site *f* normalised so the maximum value is one (cf. Sanders et al. 2011). In equation the *a_n_*s and *b_n_*s are seasonality parameters and the *c*s are the effect of air temperature (*T_A_*) on adult activity.

**S1.2 Parameter estimation**

Parameter were estimated in a Bayesian framework. The observed catch for trap *i* on the *j*th sampling occasion (*N_ij_*) is assumed to follow a negative binomial distribution, that is,

where *k* is the dispersion parameter and

$$\mu_{ij}=\sum_{t=t_{set}^{(j)}}^{t_{empty}^{(j)}} s_{i}(t)(1-\exp(-\delta_{f_{i}}(t)\Delta t))L_{i}(t),$$

is the number of midges expected to emerge during the sampling period, which is the sum of the daily numbers of midges emerging from the day on which the trap was set (*t_set_*) to the day on which it was emptied (*t_empty_*).

An exponential prior with mean 100 was used for the dispersion parameter (*k*). Gamma priors with mean 1 and shape parameter 2 were used for the density-dependence parameters (the *α*s and *β*s). Based on data on the development of *Culicoides sonorensis* larvae at different temperatures under laboratory conditions (see Chapter 2 of Wittmann (2000), reproduced in White et al. 2017), a gamma prior with mean 10 and shape parameter 5 was used for the threshold temperature (*T*_min_), while a gamma prior with mean 0.003 and shape parameter 5 was used for the development rate (*d*). Exponential priors with mean 100 were used for the productivity parameters (the *ϕ*s). Priors for parameters related to adult activity (the *a_n_*s, *b_n_*s and *c*s) were extracted from Sanders et al. (2011).

Samples from the joint posterior density were generated using an adaptive Metropolis scheme (Haario et al. 2001), modified so that the scaling factor was tuned during burn-in to ensure an acceptance rate of between 20% and 40% for more efficient sampling of the target distribution (Andrieu & Thoms 2008). Two chains of 750,000 iterations were run, with the first 250,000 iterations discarded to allow for burn-in of the chains. Each chain was subsequently thinned by selecting every 50th iteration. The adaptive Metropolis scheme was implemented in Matlab (version R2020b; The Mathworks Inc.) and the code is available online (ADD GitHub LINK). Convergence of the scheme was assessed visually and by examining the Gelman-Rubin statistic provided in the coda package (Plummer et al. 2006) in R (version 4.0.5) (R Core Team 2021).

**References**

Andrieu, C. & Thoms, J. 2008 A tutorial on adaptive MCMC. *Stat. Comput.* **18**, 343-373.

Haario, H., Saksman, E. & Tamminen, J. 2001 An adaptive Metropolis algorithm. *Bernoulli* **7**, 223-242.

Plummer, M., Best, N., Cowles, K. & Vines, K. 2006 CODA: Convergence Diagnosis and Output Analysis for MCMC. *R News* **6**, 7-11.

R Core Team 2021 R: A language and environment for statistical computing. R Foundation for Statistical Computing, Vienna, Austria. (<http://www.R-project.org/>).

White, S.M., Sanders, C.J., Shortall, C.R. & Purse, B.V. 2017 Mechanistic model for predicting the seasonal abundance of Culicoides biting midges and the impacts of insecticide control. *Parasites and Vectors* **10**, 162.

Wittman, E.J. 2000 Temperature and the transmission of arboviruses by *Culicoides* biting midges. PhD thesis, University of Bristol.
