# Supplementary figures and images for: Emergence dynamics of adult Culicoides biting midges at two farms in south-east England
Source: Parasit Vectors. 2022 Jul 11;15:251. doi: 10.1186/s13071-022-05370-z (PMC9277857; doi:10.1186/s13071-022-05370-z)

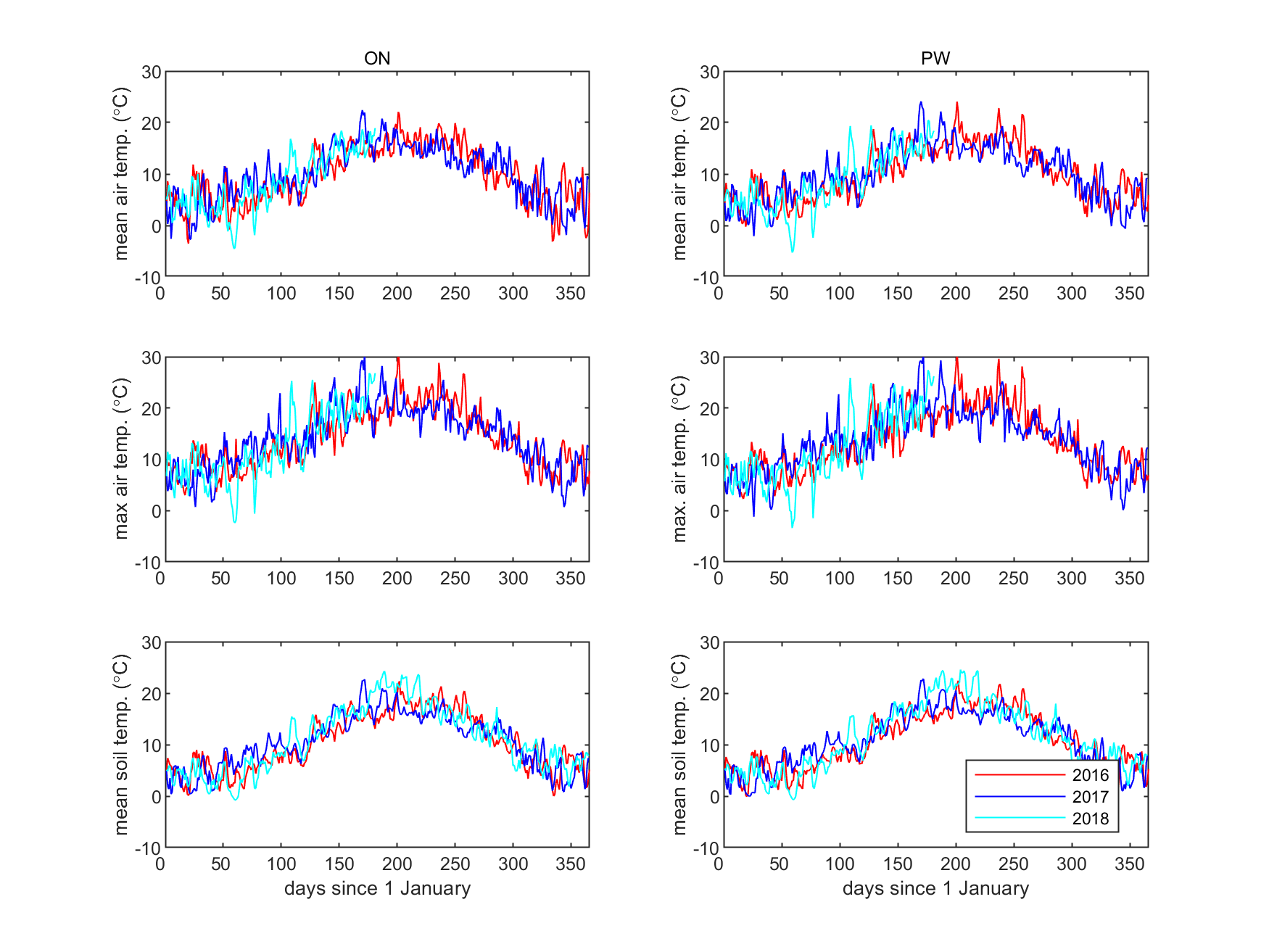

Supplement: Supplementary file 2 — Additional file 2: Figure S1. Air and soil temperature data for 2016–2018 for each farm site: daily mean and maximum air temperature (°C) (top and middle rows, respectively) and daily mean soil temperature at a depth of 3.5 cm (bottom row) for ON (left-hand column) and PW (right-hand column). Line colour indicates the year: 2016 (red); 2017 (blue); or 2018 (cyan). [file 13071_2022_5370_MOESM2_ESM.tif]

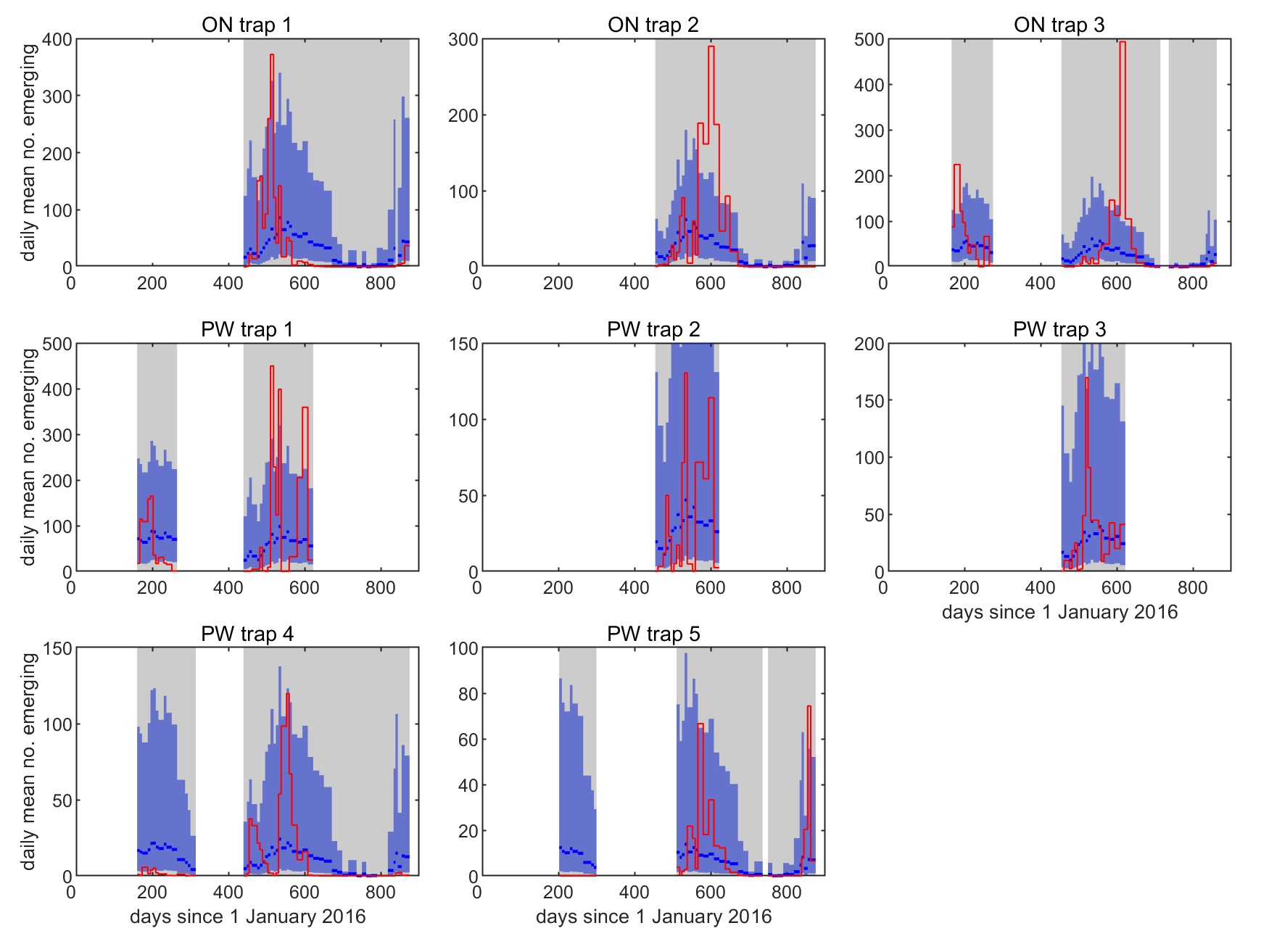

Supplement: Supplementary file 3 — Additional file 3: Figure S2. Observed and expected daily number of Culicoides biting midges caught in eight emergence traps at two farm sites in south-east England during 2016–2018. Each panel shows the observed daily mean (red) and the posterior median (blue dots) and 95% credible interval (shading) for the expected daily mean. The grey-shaded areas indicate when the samples were being collected from the trap [file 13071_2022_5370_MOESM3_ESM.tif]

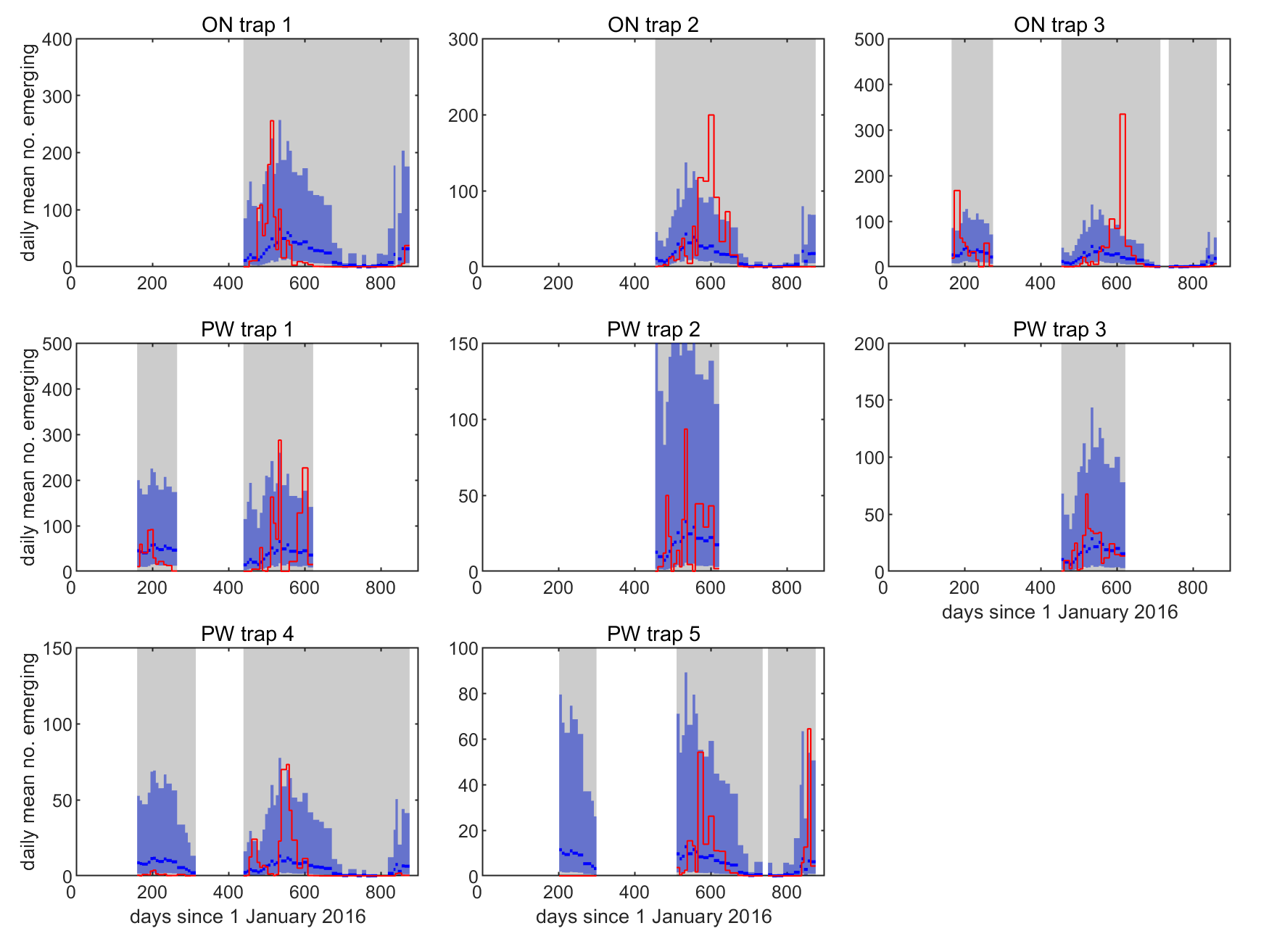

Supplement: Supplementary file 4 — Additional file 4: Figure S3. Observed and expected daily number of Culicoides obsoletus/scoticus females caught in eight emergence traps at two farm sites in south-east England during 2016–2018. Each panel shows the observed daily mean (red) and the posterior median (blue dots) and 95% credible interval (shading) for the expected daily mean. The grey-shaded areas indicate when the samples were being collected from the trap. [file 13071_2022_5370_MOESM4_ESM.tif]

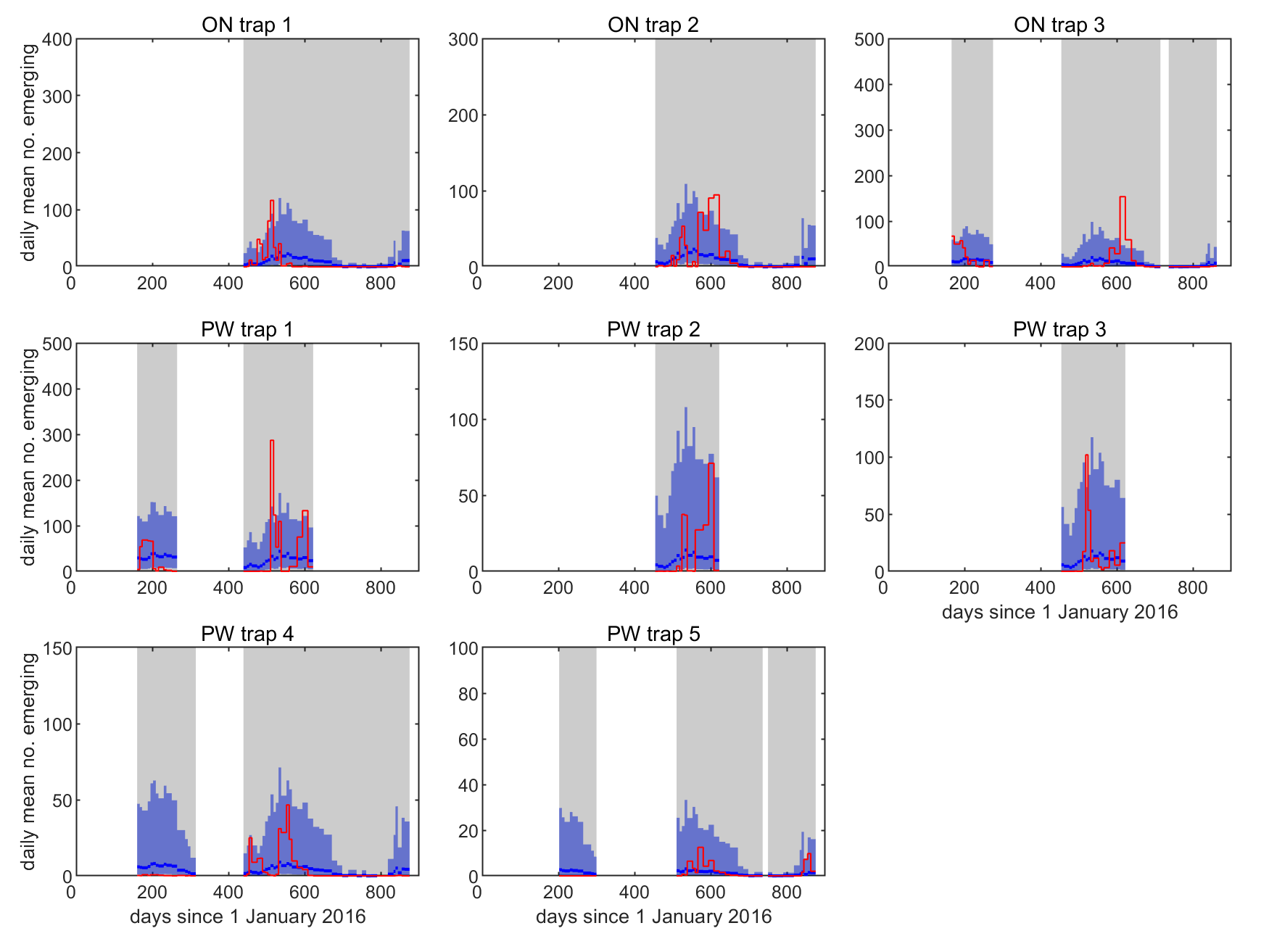

Supplement: Supplementary file 5 — Additional file 5: Figure S4. Observed and expected daily number of Culicoides obsoletus males caught in eight emergence traps at two farm sites in south-east England during 2016–2018. Each panel shows the observed daily mean (red) and the posterior median (blue dots) and 95% credible interval (shading) for the expected daily mean. The grey-shaded areas indicate when the samples were being collected from the trap [file 13071_2022_5370_MOESM5_ESM.tif]
