# Supplementary material for: Emergence dynamics of adult Culicoides biting midges at two farms in south-east England
Source: Parasit Vectors. 2022 Jul 11;15:251. doi: 10.1186/s13071-022-05370-z (PMC9277857; doi:10.1186/s13071-022-05370-z)
Supplement: Supplementary file 6 — Additional file 6: Table S1. Parameter estimates for a simple population dynamic model of pre-adult Culicoides biting midges. [file 13071_2022_5370_MOESM6_ESM.docx]

**Table S1.** Parameter estimates for a simple population dynamic model of pre-adult *Culicoides* biting midges.

| parameter | total *Culicoides* | | | *C. obsoletus/scoticus* females | | | *C. obsoletus* males | | |
| --- | --- | --- | --- | --- | --- | --- | --- | --- | --- |
|  | estimate | 95% credible limits | | estimate | 95% credible limits | | estimate | 95% credible limits | |
|  |  | lower | upper |  | lower | upper |  | lower | upper |
| trap productivity (*ϕ*) |  |  |  |  |  |  |  |  |  |
| ON, EM1 | 151.22 | 39.64 | 474.67 | 139.38 | 35.95 | 436.85 | 89.34 | 15.17 | 357.37 |
| ON, EM2 | 184.01 | 64.99 | 485.71 | 151.91 | 48.35 | 423.31 | 103.38 | 26.93 | 374.12 |
| ON, EM3 | 190.77 | 71.52 | 493.07 | 159.79 | 56.39 | 438.54 | 99.26 | 27.04 | 345.97 |
| PW, EM1 | 179.10 | 66.79 | 473.77 | 140.46 | 46.35 | 429.21 | 121.20 | 32.24 | 397.42 |
| PW, EM2 | 101.60 | 27.30 | 377.60 | 79.99 | 18.30 | 335.83 | 69.29 | 13.59 | 308.37 |
| PW, EM3 | 107.29 | 28.42 | 379.79 | 82.73 | 18.56 | 339.94 | 76.00 | 14.24 | 322.98 |
| PW, EM4 | 73.82 | 16.30 | 332.65 | 66.27 | 13.08 | 285.23 | 44.35 | 6.25 | 251.57 |
| PW, EM5 | 65.08 | 12.60 | 292.91 | 62.19 | 11.47 | 277.74 | 28.55 | 3.33 | 207.17 |
| adult activity |  |  |  |  |  |  |  |  |  |
| ON: sin, 12 mo. period (*a*_1_) | -1.56 | -1.90 | -1.22 | -1.56 | -1.89 | -1.23 | -1.56 | -1.90 | -1.23 |
| ON: cos, 12 mo. period (*b*_1_) | -3.74 | -4.71 | -2.77 | -3.75 | -4.76 | -2.77 | -3.74 | -4.73 | -2.78 |
| ON: sin, 6 mo period (*a*_2_) | -1.49 | -1.71 | -1.28 | -1.49 | -1.70 | -1.28 | -1.49 | -1.71 | -1.28 |
| ON: cos, 6 mo. period (*b*_2_) | -1.01 | -1.69 | -0.31 | -1.01 | -1.70 | -0.32 | -1.00 | -1.69 | -0.32 |
| ON: temperature (*c*) | 0.06 | 0.00 | 0.13 | 0.07 | 0.01 | 0.14 | 0.07 | 0.00 | 0.13 |
| PW: sin, 12 mo. period (*a*_1_) | -1.56 | -1.90 | -1.23 | -1.56 | -1.89 | -1.23 | -1.56 | -1.89 | -1.23 |
| PW: cos, 12 mo. period (*b*_1_) | -3.74 | -4.74 | -2.76 | -3.74 | -4.71 | -2.76 | -3.75 | -4.73 | -2.78 |
| PW: sin, 6 mo period (*a*_2_) | -1.49 | -1.71 | -1.28 | -1.49 | -1.71 | -1.27 | -1.49 | -1.71 | -1.28 |
| PW: cos, 6 mo. period (*b*_2_) | -1.00 | -1.69 | -0.32 | -0.98 | -1.67 | -0.30 | -0.99 | -1.71 | -0.29 |
| PW: temperature (*c*) | 0.03 | -0.01 | 0.08 | 0.03 | 0.00 | 0.09 | 0.04 | -0.01 | 0.10 |
| density dependence (*α*) |  |  |  |  |  |  |  |  |  |
| ON, EM1 | 0.01 | 0.00 | 0.13 | 0.01 | 0.00 | 0.15 | 0.03 | 0.00 | 0.31 |
| ON, EM2 | 0.03 | 0.00 | 0.26 | 0.04 | 0.00 | 0.31 | 0.05 | 0.00 | 0.42 |
| ON, EM3 | 0.03 | 0.00 | 0.23 | 0.04 | 0.00 | 0.27 | 0.06 | 0.00 | 0.41 |
| PW, EM1 | 0.02 | 0.00 | 0.15 | 0.02 | 0.00 | 0.22 | 0.03 | 0.00 | 0.28 |
| PW, EM2 | 0.02 | 0.00 | 0.26 | 0.02 | 0.00 | 0.28 | 0.06 | 0.00 | 0.53 |
| PW, EM3 | 0.03 | 0.00 | 0.27 | 0.03 | 0.00 | 0.34 | 0.05 | 0.00 | 0.47 |
| PW, EM4 | 0.04 | 0.00 | 0.44 | 0.08 | 0.00 | 0.61 | 0.06 | 0.00 | 0.59 |
| PW, EM5 | 0.06 | 0.00 | 0.53 | 0.07 | 0.00 | 0.58 | 0.11 | 0.00 | 0.91 |
| density dependence (*β*) |  |  |  |  |  |  |  |  |  |
| ON, EM1 | 0.21 | 0.03 | 0.65 | 0.23 | 0.03 | 0.76 | 0.27 | 0.04 | 0.84 |
| ON, EM2 | 0.22 | 0.03 | 0.68 | 0.23 | 0.03 | 0.72 | 0.25 | 0.04 | 0.76 |
| ON, EM3 | 0.21 | 0.03 | 0.68 | 0.21 | 0.03 | 0.68 | 0.25 | 0.04 | 0.81 |
| PW, EM1 | 0.21 | 0.03 | 0.67 | 0.22 | 0.03 | 0.69 | 0.23 | 0.04 | 0.67 |
| PW, EM2 | 0.22 | 0.03 | 0.72 | 0.24 | 0.03 | 0.72 | 0.28 | 0.04 | 0.84 |
| PW, EM3 | 0.23 | 0.04 | 0.73 | 0.25 | 0.04 | 0.78 | 0.26 | 0.04 | 0.82 |
| PW, EM4 | 0.27 | 0.04 | 0.88 | 0.30 | 0.05 | 0.93 | 0.33 | 0.05 | 1.02 |
| PW, EM5 | 0.30 | 0.05 | 0.88 | 0.30 | 0.05 | 0.90 | 0.40 | 0.07 | 1.10 |
| development |  |  |  |  |  |  |  |  |  |
| development rate (*d*) | 0.007 | 0.003 | 0.011 | 0.007 | 0.003 | 0.011 | 0.006 | 0.003 | 0.011 |
| threshold temperature (*T*_min_) | 5.42 | 5.16 | 5.56 | 5.47 | 5.18 | 5.60 | 5.37 | 4.76 | 5.57 |
| dispersion parameter (*k*) | 0.26 | 0.22 | 0.31 | 0.27 | 0.23 | 0.32 | 0.19 | 0.16 | 0.23 |
